# High throughput 16SrRNA gene sequencing reveals the correlation between Propionibacterium acnes and sarcoidosis

**DOI:** 10.1186/s12931-017-0515-z

**Published:** 2017-02-01

**Authors:** Meng-Meng Zhao, Shan-Shan Du, Qiu-Hong Li, Tao Chen, Hui Qiu, Qin Wu, Shan-Shan Chen, Ying Zhou, Yuan Zhang, Yang Hu, Yi-Liang Su, Li Shen, Fen Zhang, Dong Weng, Hui-Ping Li

**Affiliations:** Department of Respiratory Medicine, Shanghai Pulmonary Hospital, Tongji University, School of Medicine, 507 Zheng Min Road, Shanghai, 200433 China

**Keywords:** High throughput gene sequencing, Propionibacteria, Sarcoidosis, 16SrRNA

## Abstract

**Objective:**

This study aims to use high throughput 16SrRNA gene sequencing to examine the bacterial profile of lymph node biopsy samples of patients with sarcoidosis and to further verify the association between Propionibacterium acnes (P. acnes) and sarcoidosis.

**Methods:**

A total of 36 mediastinal lymph node biopsy specimens were collected from 17 cases of sarcoidosis, 8 tuberculosis (TB group), and 11 non-infectious lung diseases (control group). The V4 region of the bacterial 16SrRNA gene in the specimens was amplified and sequenced using the high throughput sequencing platform MiSeq, and bacterial profile was established. The data analysis software QIIME and Metastats were used to compare bacterial relative abundance in the three patient groups.

**Results:**

Overall, 545 genera were identified; 38 showed significantly lower and 29 had significantly higher relative abundance in the sarcoidosis group than in the TB and control groups (*P* < 0.01). P. acnes 16SrRNA was exclusively found in all the 17 samples of the sarcoidosis group, whereas was not detected in the TB and control groups. The relative abundance of P. acnes in the sarcoidosis group (0.16% ± 0. 11%) was significantly higher than that in the TB (Metastats analysis: *P* = 0.0010, q = 0.0044) and control groups (Metastats analysis: *P* = 0.0010, q = 0.0038). The relative abundance of P. granulosum was only 0.0022% ± 0. 0044% in the sarcoidosis group. P. granulosum 16SrRNA was not detected in the other two groups.

**Conclusion:**

High throughput 16SrRNA gene sequencing appears to be a useful tool to investigate the bacterial profile of sarcoidosis specimens. The results suggest that P. acnes may be involved in sarcoidosis development.

**Electronic supplementary material:**

The online version of this article (doi:10.1186/s12931-017-0515-z) contains supplementary material, which is available to authorized users.

## Background

Sarcoidosis is a systemic granulomatous disease and often involves the respiratory system. The disease frequently affects middle-aged adults, in particular women aged 40–50 years. The histopathology of sarcoidosis is characterized by non-caseous necrotizing granulomas, and the clinical presentation of the disease is very diverse [1]. The exact etiology and pathogenesis of sarcoidosis remain unclear although several factors, such as infection [2], genetic defects [3], immune dysfunction [4], and exposure to environmental pollutants [5], have been indicated to contribute to sarcoidosis development.

Previous reports studying the association of infection and sarcoidosis mainly focus on Mycobacterium tuberculosis (MTB) and propionibacteria, and show controversial results. Eishi et al. [6] used real-time quantitative RT-PCR to analyze bacterial DNA in the lymph node biopsy specimens of patients with sarcoidosis, TB, or other lung diseases and suggested that propionibacteria was more likely than mycobacterium to be an etiological factor of sarcoidosis. Contrarily, other reports indicate a correlation of MTB and sarcoidosis. Oswald et al. [7] found that the CD4+ and/or CD8+ T cells isolated from the bronchoalveolar lavage fluid of patients with sarcoidosis were more likely than the T cells from the control patients to be stimulated by mycobacterial ESAT-6 protein, whereas the response to P. acnes proteins was similar between the T cells of the sarcoidosis group and the T cells of the control group. Our previous studies used PCR to determine the copy number of mycobacteria and propionibacteria in the paraffin-embedded lymph node biopsy specimens of patients with sarcoidosis, TB, or other lung diseases and indicated that MTB might not be associated with sarcoidosis [8] but propionibacteria could be [9].

Most of the previous studies investigated only a few bacterial species. A full bacterial profile of the tissue specimens of patients with sarcoidosis is still lacking. The current study is filling this knowledge gap. In the current study, we aim to conduct high throughput 16SrRNA gene sequencing to investigate the bacterial profile of mediastinal lymph node specimens. The 16SrRNA gene is present in the genome of bacteria, chlamydia, rickettsia, mycoplasma, spirochaeta, and actinomycetes, but absent in eukaryotic organisms, viruses, and fungus. The sequence of V4 region of the bacterial 16SrRNA gene, which varies greatly in different bacterial species, is often considered as a unique signature for a bacterial species and thus commonly used for bacterial taxonomic classification [10–12]. The V4 region is flanked by evolutionary conserved regions. In the current study, the conserved regions were used to design PCR primers. The V4 region was then amplified by PCR with the primers and then sequenced. The sequences were analyzed and compared to the 16SrRNA gene sequence database, GreenGene database, by bioinformatics approaches to identify the bacterial taxonomic annotation [11, 13]. In the current study, we are to use bacterial 16SrRNA gene sequencing to characterize the bacterial profile of the tissue specimens of patients with sarcoidosis and identify the potential pathogenic bacteria that may correlate with the disease.

## Methods

### Tissue specimens

The protocol for collecting and handling patients’ biopsy specimens has been approved by the Institutional Review Board of Shanghai Pulmonary Hospital and Tongji University School of Medicine (Approval No: 2011-FK-10). Biopsy specimens from the mediastinal lymph node of 36 patients, who were treated in Shanghai Pulmonary Hospital between August 2014 and August 2015, were collected and analyzed. All the 36 patients, including 17 cases of sarcoidosis, 8 cases of TB, and 11 cases of control, were inpatients of Shanghai Pulmonary Hospital. The 8 patients with TB were newly diagnosed based on positive MTB in their sputum. The 11 controls included 6 cases of nonspecific lymphadenitis and 5 cases of mediastinal lymph node metastasis of lung cancer. The clinical data of the patients are displayed in Table 1. All the participating patients signed the informed consent.

**Table 1:** Patients’ clinical data

	Sarcoidosis *N* = 17	Control disease *N* = 11	Tuberculosis *N* = 8	*P* Value
Age, mean ± SD (years)	53.12 ± 7.41	53.55 ± 12.75	45.13 ± 20.73	0.3013
Male/female ratio	3/14	6/5	6/2	N/A
CXR stage: 0/I/II/III/IV number of patients (n)	0/10/4/3/0	N/A	N/A	N/A
Tuberculosis examination negative/positive (n)	17/0	N/A	0/8	N/A
BMI (kg/m^2^)	24.74 ± 2.45	22.75 ± 3.87	22.87 ± 2.34	0.1564
SACE (IU/L)	58.47 ± 26.86 (*n* = 15)	N/A	N/A	N/A
ESR (mm/h)	24.13 ± 15.82 (*n* = 16)	31.20 ± 19.81 (*n* = 6)	34.57 ± 39.43 (*n* = 7)	0.5996
24-h urinary calcium	5.70 ± 2.11(*n* = 12)	N/A	N/A	N/A
CD3 (%)	66.56 ± 8.07 (*n* = 14)	N/A	58.67 ± 15. 58 (*n* = 7)	0.1380
CD4 (%)	34. 01 ± 10.15 *(n* = 14)	N/A	35.66 ± 13.03 (*n* = 7)	0.7526
CD8 (%)	24. 87 ± 10.40 (*n* = 14)	N/A	18.99 ± 4.51 (*n* = 7)	0.1730
IgG (ng/L)	12.36 ± 2.73 (*n* = 10)	N/A	14.67 ± 7.20	0.3607
IgA (ng/L)	1.96 ± 0.56 (*n* = 10)	N/A	2.73 ± 1.12	0.0734
IgM (ng/L)	1.47 ± 0.77 (*n* = 10)	N/A	1.19 ± 0.73	0.4324
C3 (ng/L)	1.25 ± 0.20 (*n* = 10)	N/A	1.20 ± 0.29	0.6584
C4 (ng/L)	0.31 ± 0.072 (*n* = 10)	N/A	0.34 ± 0.094	0.4571

*CXR* Chest X-ray. CXR stage 0: no adenopathy and no lung infiltrates; stage I: hilar & mediastinal adenopathy only; stage II: hilar & mediastinal adenopathy plus lung infiltrates; stage III: lung infiltrates only; stage IV: Pulmonary fibrosis. BMI (Body Mass Index): weight ÷ height^2^. *SACE* serum angiotensin converting enzyme. *ESR* erythrocyte sedimentation rate. *N/A* not applicable

### Diagnostic criteria

The diagnostic criteria for sarcoidosis followed the 1999 guidelines of ATS/ERS/WASOG [1]. The histopathology of lymph node biopsy of the patients with sarcoidosis was characterized by non-caseous necrotizing granulomas and showed negative TB, fungus and/or parasitic infection, tumor, and vasculitis. Additionally, to further confirm sarcoidosis, smear-negative TB was excluded based on a negative TB-polymerase chain reaction (PCR) [8] and the comprehensive differential diagnosis scoring system for sarcoidosis and atypical TB [14]. All the 17 patients with sarcoidosis were followed up for 1 year.

Patients with TB were diagnosed according to the 2013 tuberculosis diagnosis and treatment guidelines recommended by the China Medical Association Tuberculosis Society [15]. The diagnosis of TB was based on acid-fast bacilli smear-positive sputum, positive Mycobacterium tuberculosis (M. TB) in sputum culture, positive M. TB in bronchopulmonary lavage fluid, or positive staining for acid-fast bacilli in biopsy specimens. All the patients with TB underwent anti-TB therapy and cured. The lymph node biopsy samples of lymphadenitis and lung cancer metastasis were provided by thoracic surgeons of Shanghai Pulmonary Hospital, and the diagnosis was confirmed.

### Gene sequencing of 16SrRNA

Because the sequence of V4 region of the bacterial 16SrRNA gene is a unique signature of a bacterial species, the sequence can be used to identify the specific bacterial taxonomic position [10]. The V4 region is flanked by evolutionary conserved regions, which were used to design PCR primers. The V4 region was then amplified by PCR with the primers and then sequenced. Genomic DNA was extracted from the tissue specimens using the TIANquick FFPE DNA Kit (TIANGEN, China) according to the instruction of the kit. The genomic DNA was then used as the template in the PCR to amplify the 16SrRNA gene V4 region (nucleotide position from 515 to 806). The primer sequences are: 5’-GTGCCAGCMGCCGCGGTA-3’ (forward primer) and 5’-GGACTACHVGGGTWTCTAAT-3’ (reverse primer). M stands for nucleotide A or C; H stands for nucleotide A, C, or T; V stands for nucleotide A, C, or G; W stands for nucleotide A or T [10]. Phusion® High-Fidelity PCR Master Mix (New England Biolabs, USA) was used for PCR reaction. The PCR reaction condition was: initial denaturation at 98 °C for 1 min; 30 cycles of denaturation at 98 °C for 10 s, annealing at 50 °C for 10 s, extension at 72 °C for 60 s; final extension at 72 °C for 5 min. The PCR products were electrophoresed, collected from the gel using the GeneJET Gel Extraction Kit (Thermo Scientific, USA). The purified PCR products were used to construct a DNA sequencing library using the the NEB Next® Ultra™ DNA Library Prep Kit from Illumina (San Diego, CA, USA) according to the manufacturer’s instruction. The quality of the DNA library was examined using the Quibit® 3.0 Fluorometer 2.0 (Thermo Fisher Scientific, USA) and Agilent Bioanalyzer 2100 system. The DNA library was then sequenced on the Illumina MiSeq platform (CA, USA). The collected raw sequencing data were processed according to the following steps: 1) The sequences of Barcode and primers were first removed from the raw sequencing data. 2) The sequencing data were then analyzed using the software FLASH version 1.2.7 (http://ccb.jhu.edu/software/FLASH/)[16]. For each sample, the sequencing fragments were aligned and connected to form raw sequencing tags. 3) The raw sequencing tags with low quality sequencing data were removed. 4) The final sequencing tags were compared against the Genomes OnLine Database (Gold) database (http://drive5.com/uchime/uchime_download.html) using the UCHIME Algorithm (http://www.drive5.com/usearch/manual/uchime_algo.html) [17] to identify chimeric sequences. The chimeric sequences were removed from the final sequencing tags [18]. The resulting sequences were the effective sequencing tags and used for bacterial taxonomic annotation.

### Sequence analysis

The identified 16SrRNA sequences (the effective sequencing tags) were annotated using the Uparse software package v7.0.1001 (http://drive5.com/uparse/) [19]. The annotation procedure includes the following steps: 1) Sequences that share ≥ 97% identity were considered at the same taxonomic position (belong to the same bacterial species) and assigned to one operational taxonomic unit (OTU). 2) The Uparse software automatically selected representative sequences when constructing OTUs. 3) The representative sequences were used to annotate the OTUs. OTUs were annotated with taxonomic information using the 16SrRNA gene sequence database, GreenGene database, (http://greengenes.lbl.gov/cgi-bin/nph-index.cgi) [20] and the RDP Classifier (Version 2.2, http://sourceforge.net/projects/rdp-classifier/) [21]. The sequences were annotated with taxonomic Kingdom, Phylum, Class, Order, Family, Genus, and Species. The relative abundance of each bacterial species in a sample was also determined by the software.

### Statistical analysis

To estimate if the identified 16SrRNA sequences could include all the bacteria in the samples, we conducted alpha diversity analysis. The alpha diversity was analyzed using the Quantitative Insights into Microbial Ecology (QIIME) software (Version 1.7.0) [22]. To evaluate alpha diversity, we calculated Chao index representing bacterial species abundance, number of observed species, and Shannon index representing bacterial community diversity. Rarefaction curves were plotted using the three parameters as vertical axes and the number of identified sequences as horizontal axes. Chao index, number of observed species, and Shannon index increase as the number of identified sequences increases in a sample, which indicates that unique bacterial species are increasingly identified in a sample. Saturation of the rarefaction curves indicate that the number of unique bacterial species does not increase as the number of identified sequences increases, which suggests that the identified 16SrRNA sequences may cover all the bacteria in the sample.

Bacterial relative abundance was determined to identify the most predominant bacterial species, which represent the bacterial profile in the samples. The Quantitative Insights into Microbial Ecology (QIIME) software (Version 1.7.0) and Uparse software package v7.0.1001 (http://drive5.com/uparse/) were used to analyze bacterial abundance. The Metastats software (http://metastats.cbcb.umd.edu/) was used to compare the difference in bacterial abundance between different groups. The Metastats software is specifically designed to compare bacterial abundance by quantitatively analyzing the 16SrRNA gene sequences [23].

Intra-group comparison was performed to evaluate the differences in the bacterial profile of samples in the same patient group. Inter-group comparison was performed to estimate the difference in the bacterial profile of the sarcoidosis, control, and TB patient groups. Because bacterial profile is expected to vary considerably in different individuals, evaluation of inter- and intra-group difference may reveal the unique characteristics of bacterial profile in sarcoidosis, control, and TB patient groups. For inter-and intra-group comparison of bacterial profile, R software (version 3.1.3) was used for nonparametric multi-response permutations procedure (MRPP) [24] and the analysis of similarity (Anosim) [25]. An A value (calculated from the MRPP) > 0 represented inter-group difference > intra-group difference; A < 0 represented inter-group difference < intra-group difference, *P* < 0.01 was considered statistically significant. An R value (calculated from the Anosim) > 0 represented inter-group difference > intra-group difference; R < 0 represented inter-group difference < intra-group difference; *P* < 0.01 was considered statistically significant. A *P* value was first calculated for inter-group difference in relative abundance. The *P* value was then adjusted to q value using an algorithm established by Storey and Tibshirani [26]. Based on both the *P* and q values, Taxonomic units showing significantly different relative abundance in the three groups were identified. When both *P* < 0.01 and q < 0.01, the differences were considered statistically significant. The software Graphpad prism 5 was used to prepare the curves of bacterial relative abundance. The software SPSS 20.0 was used for chi-square analysis of the inter-group difference in bacterial positive rate; *P* < 0.01 was considered statistically significant.

## Results

### Patients’ clinical data

Patients in the sarcoidosis, TB, and control groups showed similar age, body mass index, and erythrocyte sedimentation rate (Table 1). The majority of patients (*n* = 10) with sarcoidosis were at chest X-ray stage I. The percentages of specific T cell populations, including CD3, CD4, and CD8, were similar in the sarcoidosis and TB groups. So were the levels of IgG, IgA, IgM, C3, and C4 (Table 1).

### Sequence data and OTUs

Sequencing data are presented in Additional file 1: Table S1. A total of 8748 unique OTUs were constructed by the Uparse software, and an average of 1768 unique OTUs in each sample. The sequences of all the 8748 unique OTUs are displayed in Additional file 2: Table S2. The OTUs were annotated at phylum, class, order, family, genus, and species taxonomic levels using the RDP Classifier Version 2.2 (http://sourceforge.net/projects/rdp-classifier/) and the 16SrRNA sequence database, GreenGene database (http://greengenes.lbl.gov/cgi-bin/nph-index.cgi). The annotations of all the 8748 are presented in Additional file 3: Table S3. At the taxonomic genus level, a total of 545 unique bacterial genera were identified in all the samples. The relative abundances of the 545 bacterial genera in each sample are presented in Additional file 4: Table S4.

At the taxonomic genus level, the OTUs were assigned to 545 genera based on the Greengenes database (Additional file 4: Table S4). In the sarcoidosis group, 478 unique bacterial genera were identified; the top 10 most abundant genera were: Shewanella, Pseudomonas, Acinetobacter, Lactobacillus, Prochlorococcus, Bifidobacterium, Escherichia, Halomonas, Pediococcus, and Rhodococcus (Table 2). The control group showed 470 unique bacterial genera, including Bacteroides, J2-29, Prevotella, Methylobacterium, Oscillospira, [Prevotella], Roseburia, Ruminococcus, Clostridium, and Helicobacter as the top ten most abundant bacterial genera (Table 2). The TB group contained 489 unique bacterial genera, and the most abundant ones were Bacteroides, J2-29, [Prevotella], Methylobacterium, Prevotella, Clostridium, Methyloversatilis, Succinivibrio, Sutterella, and Salinispora (Table 2). All of the most abundant bacterial genera in the three patient groups belonged to the four bacterial Phylum: Proteobacteria, Firmicutes, Bacteroidetes, and Fusobacteria.

**Table 2:** The top ten most abundant bacterial genera of the three patient groups

	Sarcoidosis		Control disease		Tuberculosis	
	Bacteria	Abundance	Bacteria	Abundance	Bacteria	Abundance
1	Shewanella	7.33 ± 4.60	Bacteroides	13.13 ± 2.72	Bacteroides	13.32 ± 3.51
2	Pseudomonas	4.32 ± 2.75	J2-29	5.28 ± 3.82	J2-29	8.91 ± 2.15
3	Acinetobacter	3.31 ± 4.46	Prevotella	5.16 ± 2.63	[Prevotella]	3.25 ± 0.89
4	Lactobacillus	3.06 ± 3.98	Methylobacterium	4.11 ± 3.95	Methylobacterium	2.96 ± 2.85
5	Prochlorococcus	2.24 ± 2.83	Oscillospira	3.36 ± 1.92	Prevotella	2.75 ± 1.29
6	Bifidobacterium	1.89 ± 7.05	[Prevotella]	3.16 ± 1.02	Clostridium	2.60 ± 0.25
7	Escherichia	1.09 ± 0.83	Roseburia	2.98 ± 2.02	Methyloversatilis	1.33 ± 1.45
8	Halomonas	1.07 ± 0.71	Ruminococcus	2.65 ± 1.47	Succinivibrio	1.22 ± 0.20
9	Pediococcus	0.97 ± 1.01	Clostridium	2.56 ± 0.42	Sutterella	1.06 ± 0.27
10	Rhodococcus	0.77 ± 0.64	Helicobacter	1.07 ± 0.65	Salinispora	1.01 ± 0.37

### Alpha diversity of the sequencing data and the analyses of intra- and inter-group difference in bacterial profile

**Fig. 1:**
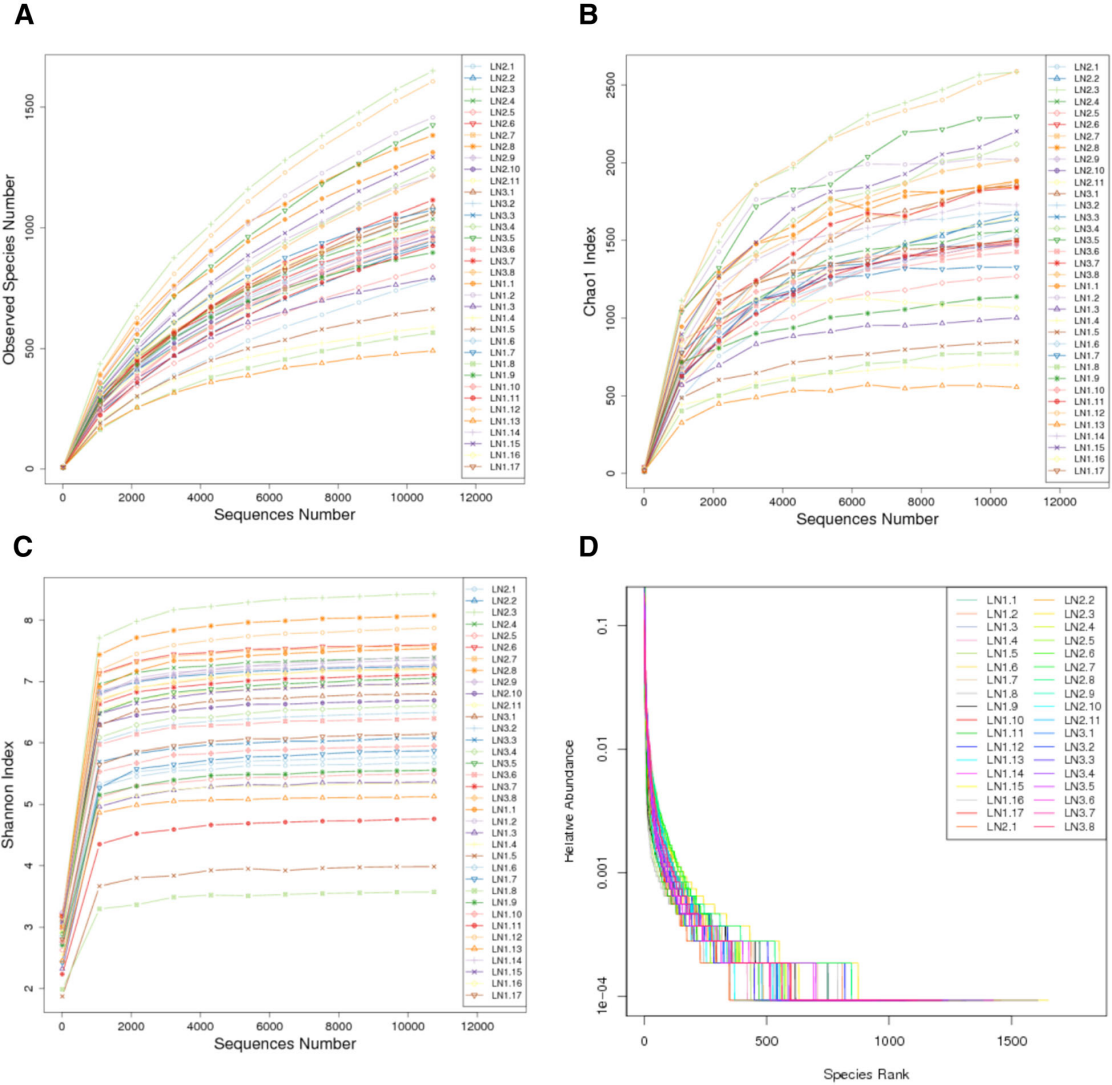
Alpha diversity. **a** Number of observed species curves. **b** Chao index curves. **c** Shannon index curves. **d** Rank abundance curves. LN1: Sarcoidosis group. LN2: Control disease group. LN3: Tuberculosis group

The rarefaction curves of number of observed species (Fig. 1a), Chao index (Fig. 1b), and Shannon index (Fig. 1c) reach a plateau, suggesting that the identified sequences may sufficiently cover the bacteria in the samples. The rank abundance curves (Fig. 1d) also become stable, indicating that species distribution is uniform. Both the Anosim and MRPP analyses revealed that the inter-group difference in bacterial profile was larger than the intra-group difference and the inter-group difference was statistically significant (All *P* < 0.01, Table 3).

**Table 3:** Inter-group analysis

Comparing pair	Anosim analysis		MRPP analysis	
	*R* value	*P* value	A value	*P* value
Sarcoidosis versus Control	0.9976	0.001	0.2817	0.001
Sarcoidosis versus Tuberculosis	0.9957	0.001	0.2772	0.001
Tuberculosis versus Control	0.4592	0.001	0.09667	0.001

### Propionibacterium was specifically detected in patients with sarcoidosis

We compared the relative abundance of each genus in the three patient groups. A total of 67 bacterial genera showed significantly higher or lower relative abundance in the sarcoidosis group compared with the TB and control groups (*P* < 0.01, q < 0.01). Among the 67 genera, 38 showed significantly lower relative abundance in the sarcoidosis group than in the TB and control groups (*P* < 0.01, q < 0.01, Table 4), and 29 showed significantly higher relative abundance in the sarcoidosis group than in the TB and control groups (*P* < 0.01, q < 0.01, Table 5).

**Table 4:** The 38 bacterial genera with significantly lower abundance in the sarcoidosis group

Bacteria Genera	Sarcoidosis (%)	Control (%)	Tuberculosis (%)	q1 value	q2 value
	*N* = 17	*N* = 11	*N* = 8		
Prevotella	0.26 ± 0.37	5.16 ± 2.63	2.75 ± 1.29	q < 0.01	q < 0.01
Clostridium	0.16 ± 0.14	2.56 ± 0.42	2.60 ± 0.25	q < 0.01	q < 0.01
Parabacteroides	0.018 ± 0.27	0.41 ± 0.13	0.25 ± 0.066	q < 0.01	q < 0.01
Bacteroides	0.12 ± 0.12	13.13 ± 2.72	13.32 ± 3.51	q < 0.01	q < 0.01
Oscillospira	0.12 ± 0.085	3.36 ± 1.92	0.58 ± 0.31	q < 0.01	q < 0.01
Methyloversatilis	0.065 ± 0.11	0.28 ± 0.15	1.33 ± 1.45	q < 0.01	q < 0.01
Methylobacterium	0.11 ± 0.065	4.11 ± 3.95	2.96 ± 2.85	q < 0.01	q < 0.01
Phascolarctobacterium	0.069 ± 0.063	0.84 ± 0.44	0.59 ± 0.20	q < 0.01	q < 0.01
Roseburia	0.055 ± 0.069	2.98 ± 2.02	0.71 ± 0.19	q < 0.01	q < 0.01
Faecalibacterium	0.050 ± 0.059	0.58 ± 0.15	0.58 ± 0.13	q < 0.01	q < 0.01
Ruminococcus	0.052 ± 0.052	0.52 ± 0.14	0.37 ± 0.059	q < 0.01	q < 0.01
Bradyrhizobium	0.054 ± 0.041	0.27 ± 0.093	0.30 ± 0.070	q < 0.01	q < 0.01
Sutterella	0.034 ± 0.037	0.87 ± 0.31	1.06 ± 0.27	q < 0.01	q < 0.01
Megamonas	0.029 ± 0.036	0.54 ± 0.20	0.68 ± 0.18	q < 0.01	q < 0.01
Prevotella	0.024 ± 0.039	3.16 ± 1.02	3.25 ± 0.89	q < 0.01	q < 0.01
Arcobacter	0.018 ± 0.022	0.13 ± 0.054	0.41 ± 0.47	q < 0.01	q < 0.01
Dialister	0.012 ± 0.014	0.076 ± 0.019	0.091 ± 0.022	q < 0.01	q < 0.01
Dorea	0.0095 ± 0.013	0.086 ± 0.057	0.047 ± 0.011	q < 0.01	q < 0.01
Allobaculum	0.0054 ± 0.014	0.36 ± 0.35	0.10 ± 0.042	q < 0.01	q < 0.01
Eubacterium	0.0043 ± 0.0089	0.035 ± 0.014	0.039 ± 0.012	q < 0.01	q < 0.01
Acetobacter	0.0059 ± 0.0071	0.025 ± 0.011	0.028 ± 0.013	q < 0.01	q < 0.01
Succinivibrio	0.0045 ± 0.0077	0.70 ± 0.30	1.22 ± 0.20	q < 0.01	q < 0.01
Mitsuokella	0.0032 ± 0.0063	0.032 ± 0.012	0.033 ± 0.0096	q < 0.01	q < 0.01
Anaerobiospirillum	0.0031 ± 0.0044	0.10 ± 0.037	0.15 ± 0.027	q < 0.01	q < 0.01
Cetobacterium	0.0033 ± 0.0041	0.077 ± 0.077	0.076 ± 0.047	q < 0.01	q < 0.01
Trichococcus	0.0017 ± 0.0045	0.040 ± 0.015	0.074 ± 0.050	q < 0.01	q < 0.01
Flavisolibacter	0.0022 ± 0.0039	0.022 ± 0.029	0.016 ± 0.0084	q < 0.01	q < 0.01
Collinsella	0.0013 ± 0.0039	0.77 ± 0.36	0.79 ± 0.18	q < 0.01	q < 0.01
Brevibacillus	0.0016 ± 0.0032	0.057 ± 0.029	0.082 ± 0.046	q < 0.01	q < 0.01
Peptococcus	0.0012 ± 0.0024	0.052 ± 0.017	0.051 ± 0.012	q < 0.01	q < 0.01
Bilophila	0.00065 ± 0.0022	0.011 ± 0.0043	0.010 ± 0.0064	q < 0.01	q < 0.01
Slackia	0.00068 ± 0.0020	0.035 ± 0.018	0.042 ± 0.018	q < 0.01	q < 0.01
J2-29	0.00065 ± 0.0019	5.28 ± 3.82	8.91 ± 2.15	q < 0.01	q < 0.01
Campylobacter	0.00051 ± 0.0016	0.056 ± 0.016	0.071 ± 0.024	q < 0.01	q < 0.01
Thermicanus	0.00026 ± 0.001	0.013 ± 0.0071	0.018 ± 0.020	q < 0.01	q < 0.01
Paraprevotella	0 ± 0	0.13 ± 0.13	0.018 ± 0.035	q < 0.01	q < 0.01
Phormidium	0 ± 0	0.011 ± 0.018	0.0089 ± 0.012	q < 0.01	q < 0.01
Sporolactobacillus	0 ± 0	0.0093 ± 0.019	0.0074 ± 0.0038	q < 0.01	q < 0.01

q1 value: Sarcoidosis versus Control q value. q2 value: Sarcoidosis versus Tuberculosis q value

**Table 5:** The 29 bacterial genera with significantly higher abundance in the sarcoidosis group

Bacteria Genera	Sarcoidosis (%)	Control (%)	Tuberculosis (%)	q1 value	q2 value
	*N* = 17	*N* = 11	*N* = 8		
Shewanella	7.33 ± 4.60	0.014 ± 0.015	0.013 ± 0.011	q < 0.01	q < 0.01
Pseudomonas	4.32 ± 2.75	0.12 ± 0.052	0.17 ± 0.080	q < 0.01	q < 0.01
Prochlorococcus	2.24 ± 2.83	0.021 ± 0.034	0.090 ± 0.096	q < 0.01	q < 0.01
Paenibacillus	0.59 ± 2.14	0.012 ± 0.0095	0.010 ± 0.0069	q < 0.01	q < 0.01
Pediococcus	0.97 ± 1.01	0.088 ± 0.095	0.046 ± 0.022	q < 0.01	q < 0.01
Halomonas	1.07 ± 0.71	0.0038 ± 0.0041	0.0053 ± 0.0047	q < 0.01	q < 0.01
Rhodococcus	0.77 ± 0.64	0.012 ± 0.0088	0.011 ± 0.0056	q < 0.01	q < 0.01
Candidatus Portiera	0.46 ± 0.42	0.0029 ± 0.0032	0.0059 ± 0.0037	q < 0.01	q < 0.01
Alteromonas	0.34 ± 0.30	0.0032 ± 0.0036	0.0030 ± 0.0052	q < 0.01	q < 0.01
Idiomarina	0.034 ± 0.59	0.00045 ± 0.0010	0.00033 ± 0.00094	q < 0.01	q < 0.01
Marinomonas	0.30 ± 0.27	0.00020 ± 0.00066	0.00064 ± 0.0012	q < 0.01	q < 0.01
Vibrio	0.21 ± 0.24	0.023 ± 0.011	0.033 ± 0.015	q < 0.01	q < 0.01
Corynebacterium	0.25 ± 0.15	0.075 ± 0.051	0.052 ± 0.011	q < 0.01	q < 0.01
Tolumonas	0.18 ± 0.21	0.0013 ± 0.0019	0.067 ± 0.12	q < 0.01	q < 0.01
HTCC	0.19 ± 0.17	0.0047 ± 0.0041	0.016 ± 0.024	q < 0.01	q < 0.01
Glaciecola	0.13 ± 0.20	0.0016 ± 0.0021	0.0011 ± 0.0015	q < 0.01	q < 0.01
Aliivibrio	0.10 ± 0.20	0.0071 ± 0.012	0.0050 ± 0.0043	q < 0.01	q < 0.01
Swaminathania	0.14 ± 0.15	0.00020 ± 0.00066	0.0011 ± 0.0015	q < 0.01	q < 0.01
Propionibacterium	0.16 ± 0.11	0 ± 0	0 ± 0	q < 0.01	q < 0.01
Micrococcus	0.10 ± 0.16	0.015 ± 0.0071	0.014 ± 0.0071	q < 0.01	q < 0.01
Thalassobius	0.11 ± 0.12	0.013 ± 0.010	0.0073 ± 0.0038	q < 0.01	q < 0.01
Saccharopolyspora	0.096 ± 0.11	0.0028 ± 0.0040	0.0045 ± 0.012	q < 0.01	q < 0.01
Ochrobactrum	0.069 ± 0.056	0.0067 ± 0.0053	0.0097 ± 0.011	q < 0.01	q < 0.01
Weissella	0.060 ± 0.042	0.0038 ± 0.0046	0.0033 ± 0.0038	q < 0.01	q < 0.01
MSBL3	0.050 ± 0.051	0.00021 ± 0.00069	0.00063 ± 0.0012	q < 0.01	q < 0.01
SGSH944	0.039 ± 0.052	0.0013 ± 0.0041	0.00033 ± 0.00093	q < 0.01	q < 0.01
Methylophaga	0.032 ± 0.046	0.0025 ± 0.0040	0.0019 ± 0.0018	q < 0.01	q < 0.01
Thauera	0.029 ± 0.048	0.0011 ± 0.0022	0.0033 ± 0.0052	q < 0.01	q < 0.01
Xanthobacter	0.015 ± 0.013	0.00045 ± 0.00099	0.0013 ± 0.0020	q < 0.01	q < 0.01

q1 value: Sarcoidosis versus Control q value. q2 value: Sarcoidosis versus Tuberculosis q value

Among the 29 bacterial genera with significantly higher relative abundance in the sarcoidosis group, The Propionibacterium Genus was found in all the 17 patients with sarcoidosis (100% positive rate) but was completely absent in all the 8 patients with TB (0% positive rate, *χ*2 = 25, *P* < 0.0001) and 11 control patients (0% positive rate, *χ*2 = 28, *P* < 0.0001). In contrast, the other 28 bacterial genera were detected in all the patients in the three groups, suggesting that these bacterial genera may not be specific for patients with sarcoidosis. The relative abundance of the Propionibacterium Genus in the sarcoidosis group was 0.16% ± 0. 11%, which was significantly higher than that in the control (0%, *P* = 0.001, q = 0.0055) and TB groups (0%, *P* = 0.0010, q = 0.0063).

In addition to propionibacteria, mycobacteria [7, 8, 27, 28], Borrelia [29, 30], mycoplasma [31], and chlamydia [32] have also been investigated as pathogenic organisms. Thus, we examined the four microorganisms. Mycobacterium was detected in the three patient groups but showed very low relative abundance. Although the relative abundance of Mycobacterium in the TB group (0.077% ± 0.037%) was higher than that in the sarcoidosis (0.056% ± 0.029%, *P* = 0.18, q = 0.38) and control (0.052% ± 0.073%, *P* = 0.36, q = 0.94) groups, the differences was not statistically significant. The relative abundance of mycoplasma was also very low in the three groups: 0.0067% ± 0.0090% in the sarcoidosis group, 0.0026% ± 0.0034% in the control group (*P* = 0.59, q = 0.77), and 0.0020% ± 0.0023% in the TB group (*P* = 0.19, q = 0.39). The differences were not statistically significant. Neither Borrelia nor and chlamydia was detected in the samples.

**Fig. 2:**
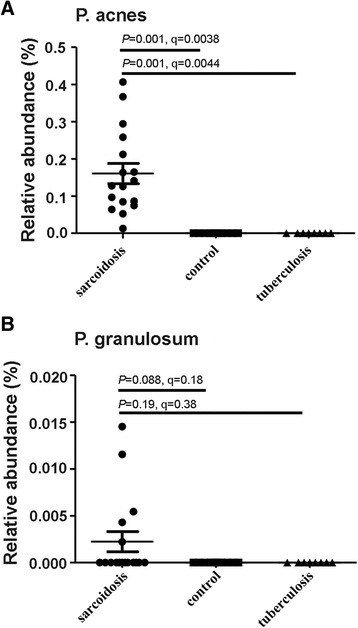
Comparison of relative abundance of P. acnes and P. granulosum in the three patient groups. **a** Relative abundance of P. acnes. **b** Relative abundance of P. granulosum

**Fig. 3:**
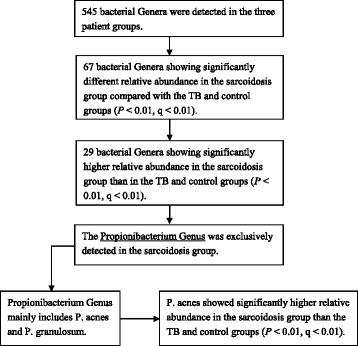
Identification of the unique bacteria in the sarcoidosis group

The two predominant bacterial species in the Propionibacterium Genus are Propionibacterium acnes (P. acnes) and Propionibacterium granulosum (P. granulosum). P. acnes was detected in all the 17 patients with sarcoidosis, whereas P. granulosum was detected in only 5 of the 17 patients. Both Propionibacterium species were not found in the control and TB groups. The relative abundance of P. acnes in the sarcoidosis group (0.16% ± 0. 11%) was significantly higher than that in the control (0%, *P* = 0.0010, q = 0.0038) and TB (0%, P = 0.0010, q = 0.0044) groups (Fig. 2a). In contrast, the relative abundance of P. granulosum in the sarcoidosis group (0.0022% ± 0. 0044%), which was extremely lower than the relative abundance of P. acnes, was not significantly different compared with that in the control (0%, *P* = 0.088, q = 0.18) and TB (0%, *P* = 0.19, q = 0.38) groups (Fig. 2b). These data indicate that P. acnes may be more likely to correlate with sarcoidosis than other bacteria. The process of identification of the unique bacterium in the sarcoidosis group is displayed in Fig. 3.

## Discussion

The association of infection and sarcoidosis etiology has been studied extensively. Molecular biological approaches have been used to search bacterial genome in sarcoidosis lesions [33]. Previous studies have shown that possible pathogenic organisms for sarcoidosis include propionibacteria [8, 33, 34], mycobacterium [7, 8, 27, 28], borrelia [29, 30], mycoplasma [31], and chlamydia [32]. Particularly, MTB and propionibacteria are the most frequently reported bacteria correlating with sarcoidosis [35, 36]. In spite of the efforts, no consistent and definitive conclusion has been drawn. Piotrowski et al. reported that MTB infection triggered a sarcoid reaction, and thus they proposed that TB and sarcoidosis might be different clinical manifestations of MTB infection [28]. However, our previous study, which determined the copy number of MTB in lymph node specimens of patients with TB or sarcoidosis, suggests that MTB may not be the pathogenic bacterium of sarcoidosis [8].

Propionibacteria are gram positive anaerobic bacteria and belong to the normal flora of the skin. Since Abe et al. first isolated P. acnes from lymph node biopsy specimens of patients with sarcoidosis in 1984 [34], P. acnes has been suspected to be a pathogenic bacterium for sarcoidosis. Negi et al. used P. acnes-specific monoclonal antibodies that react with cell-membrane-bound lipoteichoic acid (PAB antibody) in immunohistochemistry to locate P. acnes in the lung tissue and lymph node specimens of patients with sarcoidosis, and they discovered that the PAB antibodies specifically bound to the sarcoidosis specimens but did not react with tissues from patients with non-sarcoidosis disease [33]. We previously performed a meta-analysis to systematically review the articles that focused on the association of P. acnes and sarcoidosis [37], and our results support that P. acnes is highly likely a pathogen of sarcoidosis.

On the other hand, other studies indicate that P. acnes may not be associated with sarcoidosis. Ishige et al. found that 56% – 73% of specimens (lung tissues: 24/43, mediastinal lymph node specimens: 8/11) from patients with non-sarcoidosis diseases showed positive P. acnes culture [38]. Oswald et al. discovered that compared with the CD4+ and/or CD8+ T cells from the controls, the T cells from patients with sarcoidosis reacted to mycobacterial ESAT-6 protein more frequently but showed similar response to P. acnes proteins [7]. They also used matrix-assisted laser desorption ionization imaging mass spectrometry to locate the ESAT-6 protein and the P. acnes proteins in the tissue specimens and found that ESAT-6 signals specifically existed in sarcoidosis granulomas, whereas P. acnes signals were distributed non-specially in the tissues [7]. These studies suggest that P. acnes may be not involved in sarcoidosis etiology.

To identify potential pathogenic bacteria of sarcoidosis, we previously used quantitative PCR to quantify the copy number of 16SrRNA gene of P. acnes and P. granulosum in the lymph node specimens and found that propionibacteria existed in 80% of sarcoidosis specimens but only in 4.5% of TB specimens [9]. To further confirm our previous conclusions, in the current study, we used high throughput 16SrRNA gene sequencing to investigate the bacterial profile of lymph node specimens of sarcoidosis, TB, or other lung diseases. Our results showed that P. acnes were exclusively present in sarcoidosis samples, and both P. acnes and P. granulosum were not detected in the TB and control groups. These findings clearly support that P. acnes is associated with sarcoidosis.

The possible pathogenic mechanism of P. acnes in sarcoidosis development has been tested in animal models. Kouji Iio et al. used heat-inactivated P. acnes and Freund's complete adjuvant to induce pulmonary granuloma in mice, and found that the total number of lymphocytes and the ratio of CD4+ to CD8+ T cells in the bronchoalveolar lavage fluid of the mice with pulmonary granuloma were higher than those of the control mice [39]. Their study suggests that P. acnes may cause pulmonary allergic inflammation, which may consequently lead to granuloma. Eishi et al. also proposed that P. acnes could cause sarcoidosis by an allergic endogenous infection [40]. P. acnes or the bacteria-related antigens may interact with the pattern recognition receptors to stimulate macrophages and T cells to release pro-inflammatory factors, such as IFN-γ, IL-2, TNF-α, and IL-6, which ultimately disrupts the immune homeostasis in patients and induces sarcoidosis development [36]. Thus, P. acnes may cause sarcoidosis by interfering in the immune homeostasis.

Compared with quantitative PCR approach, high throughput16SrRNA gene sequencing is a more effective approach to investigate bacterial profile of biopsy specimens and identify the predominant bacterial species in the specimens. The sequencing approach also allows us to compare the diversity and relative abundance of bacteria in specimens. Although quantitative PCR approach may be sensitive, low-cost, and fast, the sequencing approach can produce data for comprehensive analyses, such as bacterial taxonomy, bacterial diversity, and the correlation of bacterial profile evolution and sarcoidosis progression. The current study first used high throughput sequencing approach to investigate the bacterial profile in lymph node specimens of patients with sarcoidosis and to identify that propionibacteria were exclusively present in the sarcoidosis specimens. The advantages of the sequencing method include high throughput, high reproducibility, and high accuracy. The limitations of the sequencing method include technically complex and requiring special instrument and software for the analysis. In addition, the roles of those bacteria with significantly higher or lower abundance in the sarcoidosis group compared with the control and TB groups in the development of sarcoidosis remain unclear. Furthermore, the control groups in the current study included lymph node specimens of patients with nonspecific lymphadenitis and of patients with mediastinal lymph node metastasis of lung cancer, which may contribute to the highly various bacterial profiles in the control group. Another limitation of the current study is that the number of tissue specimens in the current study was relatively small. We are currently collecting more samples to further investigate the dynamic evolution of bacterial profiles in the sarcoidosis lesions at different disease stages.

## Conclusions

The current study used high throughput 16SrRNA gene sequencing to demonstrate that P. acnes exclusively existed in the mediastinal lymph node specimens of patients with sarcoidosis but was absence in the specimens of patients with TB or other non-infectious lung diseases. These findings indicate that P. acnes may correlate with sarcoidosis etiology.

## Additional files

Additional file 1: Table S1. Sequencing data of the 36 tissue speciments. (XLS 15 kb)
Additional file 2: Table S2. Sequences of the unique OTUs. (XLS 3616 kb)
Additional file 3: Table S3. Taxonomic annotation of the unique OTUs. (XLS 1114 kb)
Additional file 4: Table S4. Relative abundance of the 545 bacterial genera in each sample. (XLS 330 kb)

## Abbreviations

Anosim: The analysis of similarity
MRPP: Multi-response permutations procedure
MTB: Mycobacterium tuberculosis
OTU: Operational taxonomic unit
P. acnes: Propionibacterium acnes
P. granulosum: Propionibacterium granulosum
PCR: Polymerase chain reaction.
QIIME: Quantitative insights into microbial ecology
TB: Tuberculosis
